# Diverse conditions support near-zero growth in yeast: Implications for the study of cell lifespan

**DOI:** 10.15698/mic2019.09.690

**Published:** 2019-08-20

**Authors:** Jordan Gulli, Emily Cook, Eugene Kroll, Adam Rosebrock, Amy Caudy, Frank Rosenzweig

**Affiliations:** 1 School of Biological Sciences, Georgia Institute of Technology, Atlanta, GA 30332.; 2 Donnelly Centre for Cellular and Biological Research and Department of Molecular Genetics, University of Toronto, Toronto, Ontario, Canada.; 3 Present address: Stony Brook School of Medicine, Stony Brook University, Stony Brook, NY 11794.

**Keywords:** baker's yeast, chronological lifespan (CLS), near-zero growth, starvation, giant yeast colonies, retentostats, encapsulation, immobilized cell reactors

## Abstract

Baker's yeast has a finite lifespan and ages in two ways: a mother cell can only divide so many times (its replicative lifespan), and a non-dividing cell can only live so long (its chronological lifespan). Wild and laboratory yeast strains exhibit natural variation for each type of lifespan, and the genetic basis for this variation has been generalized to other eukaryotes, including metazoans. To date, yeast chronological lifespan has chiefly been studied in relation to the rate and mode of functional decline among non-dividing cells in nutrient-depleted batch culture. However, this culture method does not accurately capture two major classes of long-lived metazoan cells: cells that are terminally differentiated and metabolically active for periods that approximate animal lifespan (e.g. cardiac myocytes), and cells that are pluripotent and metabolically quiescent (e.g. stem cells). Here, we consider alternative ways of cultivating *Saccharomyces cerevisiae* so that these different metabolic states can be explored in non-dividing cells: (i) yeast cultured as giant colonies on semi-solid agar, (ii) yeast cultured in retentostats and provided sufficient nutrients to meet minimal energy requirements, and (iii) yeast encapsulated in a semisolid matrix and fed *ad libitum* in bioreactors. We review the physiology of yeast cultured under each of these conditions, and explore their potential to provide unique insights into determinants of chronological lifespan in the cells of higher eukaryotes.

## INTRODUCTION

Aging, or the progressive loss of function over time, is a hallmark feature of all living cells, including ‘simple' organisms such as baker's yeast [1]. More than half a century ago, Mortimer and Johnson [2] reported that individual yeast cells are mortal and have a limited capacity for cell division. Subsequent demographic analyses showed that mortality increased exponentially as yeast populations underwent successive rounds of replication in batch culture. Replicatively aging yeast undergo characteristic morphological and biochemical changes that include increasing cell size, accumulation of bud scars, slowing of the cell cycle, and accumulation of extrachromosomal rDNA circles [3–5]. Genetic variation is associated with strain-specific differences in the rate of functional decline, manifest as strikingly different mean replicative lifespans (RLS) among wild and laboratory yeasts [6, 7]. Yeast also age chronologically, as evidenced by the decline and eventual death of a non-dividing cell over its chronological lifespan (CLS). Because microbes cannot divide in the absence of essential nutrients, yeast CLS has most frequently been studied in relation to the survival of free-floating (planktonic) cells in nutrient-depleted liquid culture [8–10]. Such cells enter G_0_ arrest and initially undergo metabolic and structural changes that include accumulation of storage carbohydrate, cell wall thickening, an overall decline in protein synthesis, an increase in stress tolerance, and a shift to respiratory metabolism [8, 11]. Viability among starving planktonic yeast cells diminishes over time, as does their replicative capacity, suggesting that RLS and CLS may be linked mechanistically [11]. Caloric restriction (CR) during the time non-dividing yeast age delays the progressive reduction in RLS [12–14]. Indeed, among chronologically old cells, those that have the lowest mitochondrial membrane potential (reducing ATP production) [15] also have the longest subsequent RLS; thus, mitochondrial function may constitute a causal link between CLS and RLS [16]. Further evidence for this link is provided by rho(0) cells, which experience both lower mitochondrial membrane potential and longer RLS than do rho(+) cells [17]. Strain-specific variation in CLS exists among wild yeast isolates, assayed as survivorship of starving planktonic cells [18], and the genetics underlying this variation has been generalized to other eukaryotes, including animals [19].

## CHRONOLOGICAL AGING IN STARVED PLANKTONIC CULTURES

Screens for CLS mutants in starved yeast cultures have uncovered genes involved in stress-resistance and nutrient-signaling pathways [20–23]. Some of these pathways, like the target-of-rapamycin (TOR)-pathway, are highly conserved among eukaryotes and have been implicated in aging processes in worms, flies and mammals [24–27]. However, starvation poorly mimics the physiology of major classes of metazoan cells. Terminally differentiated cells such as cardiac myocytes and neurons consume a large proportion of the organism's energy and are typically well nourished [28]. Although growth-arrested, such cells remain metabolically active and perform large amounts of work with minimal functional decline, often for decades in higher animals [29]. Starvation also poorly mimics the physiology of mitotically quiescent cells, such as hematopoietic stem cells and myosatellite cells that, respectively, can quickly be recruited to form blood cells or to regenerate injured skeletal muscle. Satellite cells are “lying low but ready for action,” being not only mitotically but also metabolically quiescent, having few mitochondria, and deriving most of their maintenance energy requirements from anaerobic (i.e. fermentative) metabolism [30]. Hematopoietic stem cells also appear to derive most of their maintenance energy requirements from fermentative metabolism, and indeed, the switch to mitochondrial respiration appears to be necessary for stem cell differentiation [31, 32].

**Figure 1:**
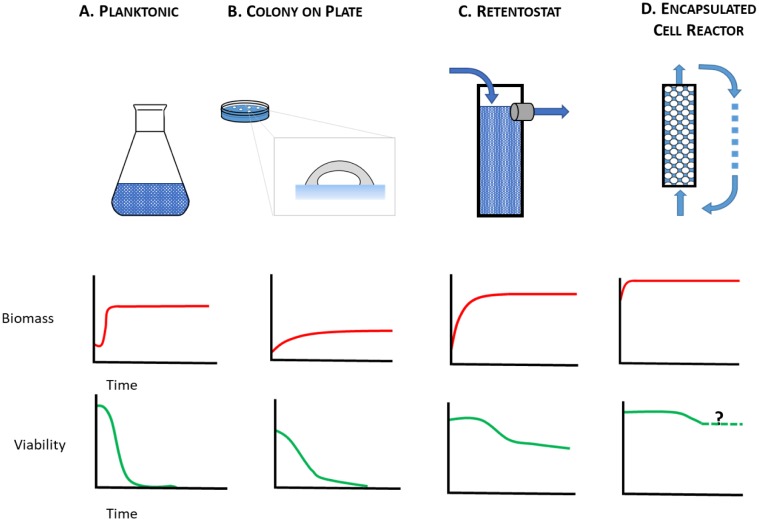
FIGURE 1: Chronological lifespan of non-dividing yeast has been studied using multiple culture methods. **(A)** planktonic cultures, **(B)** colonies on plates, **(C)** retentostats, and **(D)** encapsulated cell reactors, each of which presents a different time-dependent profile of biomass accumulation and cell viability.

The starving yeast aging paradigm, where lifespan is measured in nutrient-depleted planktonic cells, therefore falls short as a model to study the physiology of cells that are either growth-arrested but metabolically active, or growth-arrested but metabolically quiescent, i.e. those cells that are on the front lines and those that are held in reserve. To make up for this shortfall, alternative yeast culture conditions are needed in which mitotically-arrested cells can be studied under circumstances that range from caloric excess to CR. Here, we consider alternatives to the starving yeast paradigm **(Figure 1A)** where near-zero growth rates can be achieved by other means. These include aging yeast cultured on solid medium **(Figure 1B)**, in retentostats **(Figure 1C)**, or as encapsulated cells in continuously-fed bioreactors **(Figure 1D)**. With these culture methods at their disposal, yeast researchers can investigate determinants of chronological lifespan in a variety of contexts: closed systems **(Figure 1A, B)**, and open systems **(Figure 1C, D)**, matrix-free **(Figure 1 A, C)**, and matrix-associated **(Figure 1B, D)**, nutrient-limited **(Figure 1A, B, C)**, and nutrient-replete **(Figure 1D)**. Below we consider how these different contexts lead to physiological states that better approximate metabolism in non-dividing animal cells than do starving planktonic yeast.

## CHRONOLOGICAL AGING IN YEAST COLONIES

### General considerations

Yeast CLS has been studied in the context of colonies growing on the surface of nutrient agar [33, 34], typically under aerobic conditions on complex media [35, 36] for periods ranging from 20-130+ days. Unlike cells cultured in ideally-mixed liquid media, giant colonies (created by spotting a cell suspension onto GMA [37]) are in a spatially-structured environment, where they experience gradients of nutrients, waste products, signaling molecules, and gases [38]. These gradients, in turn, influence how cells stratify in colonies and age within those strata, much like a morphogenetic field in early embryological development [39]. By ten days post-inoculation, colonies have differentiated vertically into two cell types, only one of which divides [40]. In this respect, the colony resembles a multicellular organism, in that it is largely clonal and consists of a mixed population of dividing and non-dividing cells.

### Cells in a colony display slower growth, and higher viability than aging planktonic cells

The various gradients experienced by cells in colonies [41] create selective pressures not experienced by planktonic cells in liquid culture. Cells on agar and cells in liquid culture behave similarly just after inoculation [36]. But soon thereafter colonies undergo a longer period of slow growth than do planktonic cells (>8 days on solid medium vs. 40 hours in liquid) [42]. Diffusion provides cells growing on solid agar with a slow, steady nutrient supply [40], making it possible for the population to gradually expand for up to 32 days [35]. However, not every cell in a colony can divide: the number of colony-forming units (CFUs) recovered after dilution and plating on fresh agar asymptotes after 18 days. And by 90 days as much as 25% of a colony consists of cells that are alive but unable to reproduce on rich medium, a likely result of chronic nutrient deprivation, much like oligotrophic environments in nature host large numbers of viable but nonculturable cells [43].

Patterns of reproduction and survivorship in giant colonies contrast sharply with those patterns in planktonic culture, where biomass accumulation typically ceases 3 days post-inoculation [44]. After 10 days in synthetic dextrose complete (SDC; 2% dextrose) medium, only 5% of starving planktonic yeast may be viable [45]. By contrast, after 10 days on GM agar (1% yeast extract, 3% glycerol, 1.5% agar), yeast in giant colonies are 90% viable and do not fall to 5% until after 135 days. In both instances, viability was estimated as the proportion of total direct counts recovered as CFUs [35]. At all time-points measured, yeast in colonies exhibit greater viability than their isogenic counterparts in planktonic culture, even among strains that differ in CLS [35, 46, 47]. This observation has led Palkova and colleagues to speculate that prolonged slow growth of yeast cells in colonies is analogous in some ways to CR [35], a factor shown to increase lifespan in all species examined to date [12, 14, 48, 49].

### Yeast in aging colonies become physiologically-differentiated

**Figure 2:**
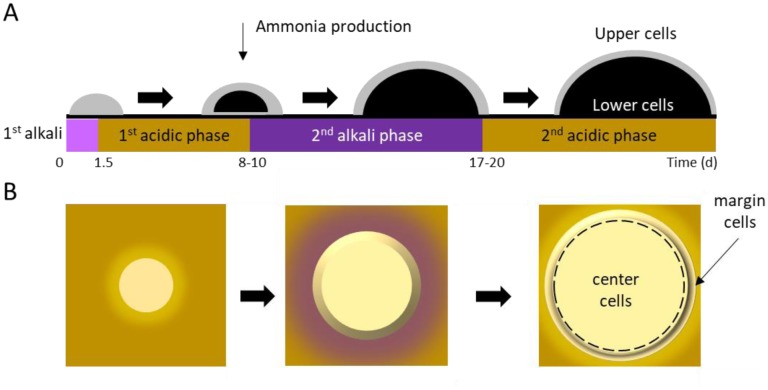
FIGURE 2: Yeast colonies transition through alkali (pH ~6.8) and acidic (pH ~5.2) phases, causing the area around a colony to change in color from yellow to purple and back again on agar containing Bromocresol purple. The pH changes contribute to cellular differentiation in both the vertical (A) and horizontal (B) dimensions. **Gray** indicates healthy, dividing cells and **black** indicates non-dividing cells.

In an aging colony, subpopulations of cells differentiate in both vertical (**Figure 2A**; upper vs. lower) and horizontal (**Figure 2B**; center vs. margin) dimensions, with subpopulations aging in different ways and at different rates [50, 51]. Following 10 days of incubation, spatially-segregated cell types can be distinguished from one another and from their progenitors with respect to morphology, nutrient utilization, and stress resistance. Cellular differentiation within giant colonies has been linked to production of volatile ammonia, which is believed to contribute to, or even drive, formation of stratified layers within a colony. The process has been described in detail [52–55], and recently reviewed by Vachova and Palkova [50, 51].

### Vertical stratification of U and L cells in aging yeast colonies

Along the vertical axis, cells in giant aging colonies stratify into upper (U) cells and lower (L) cells. U cells typically exhibit multiple small vacuoles and swollen mitochondria having few cristae [38]. Further characteristics of U cells include glycogen accumulation, lipid droplet formation, and low mitochondrial transmembrane potential, as indicated by staining with DiOC_6_, a mitochondrial dye. In contrast with U cells, L cells contain numerous mitochondria with well-developed cristae, and exhibit a high transmembrane potential by DiOC_6_ staining, [36, 56]. Accordingly, L cells produce 3-fold more reactive oxygen species than U cells, and exhibit higher respiratory capacity [56].

Paradoxically, L cells display attributes reminiscent of both starved and exponentially-growing planktonic cells. Like starved planktonic cells, L cells are actively respiring: glucose-repressible genes are de-repressed, including those in mitochondrial biogenesis, oxidative phosphorylation, and nonfermentable carbon source utilization (e.g. *HAP5, USV1, RTG1*) [56, 57]. However, like exponential-phase planktonic cells, L cells are sensitive to heat shock, cell wall dissolution by zymolyase, and exhibit no TOR activity [56]. Further, recent studies on finely-dissected giant colonies indicate that among L-cells, upper and lower L subpopulations can be discriminated on the basis of their distinctive patterns of gene expression [57].

Physiological differences between U and L cells lead to differences in stress resistance and CLS within an aging colony. For example, at day 50 only 10% of L cells are still viable, compared to 50% of U cells [50]. Higher viability among U cells is correlated with activation of regulatory pathways like TOR, typical of cells growing under nutrient-rich conditions [58]. But U cells also exhibit features typical of yeast under nutrient limitation, such as activation of the general amino acid control pathway [50]. This unusual physiology suggests active consumption of some nutrients, but limitation by others, and contrasts with the physiology of L cells, which upregulate genes involved in nonfermentable carbon source utilization rather than TOR.

### Cell stratification benefits some cell types at the expense of others, contributing to colony longevity

Autophagy is important for survival in U, but not L, cells. Colonies derived from strains having defects in autophagy (*atg1*Δ*, atg12*Δ*, atg5*Δ*, atg8*Δ) exhibit reduced U cell viability, particularly in older colonies, but have no effect on L cell viability. This observation raises the possibility that L cells may provide an energy source for U cells. Indeed, L cells activate degradative pathways related to proteasomal and vacuolar function, and upregulate expression of genes involved in glucose export [50]. Defects in these genes diminish U cell viability, providing evidence that U cells depend on L cell degradation and L cell glucose export for their survival. Ninhydrin staining indicates that L cells also release amino acids, with their total intracellular amino acid content decreasing by 23% between days 15 and 20. Finally, L cells are zymolyase-sensitive and upregulate cell wall glucanases (Scw11p, Dse4p, Egt2p), suggesting they may release cell wall constituents that serve as carbon sources for U cells. The consumption of L cells by U cells may benefit the colony as a whole, assuring not only its survival, but also its dispersal. All things being equal, cells on a colony's exterior are more likely to be dispersed either by water or by insects than cells in its interior. However, exterior cells are distant from diffusible nutrients in the colony substratum. L cells may thus engage in a form of altruism that benefits their kin, as U and L cells are essentially clones of one another [59].

Horizontally-stratified cells may also engage in autophagy. Cells in the colony center begin to differentiate from those on the colony margins around the same time that U and L cells differentiate **(Figure 2)**. Superoxide concentrations increase in center cells, even as they simultaneously decrease in margin cells [54, 60]. As a result, center cells undergo accelerated programmed cell death (PCD) relative to margin cells. In contrast with either accidental or regulated cell lysis, PCD results in the release of lytic enzymes that damage or destroy healthy neighboring cells [50, 51]. Thus, similar to U cells receiving nourishment from L cells, center cells' PCD may contribute to overall colony survival by providing nutrients that nourish margin cells. This idea is supported by the observation that removing center cells from a differentiated colony leads to diminished growth at the colony margin [46]. While cells in a giant yeast colony undergo apoptosis similar to cells undergoing PCD in a multicellular organism, the process does not recruit Mca1p or Aif1p, and must therefore be regulated differently than in metazoans [36].

### Giant colonies as a model to study aging and cancer

Yeast in a colony ages differently than starving, planktonic yeast. As planktonic cells age, they increasingly express stress-defense genes (*SOD1, SOD2, CTT1*) [61, 62]. But as colonies age, expression of stress-related genes, including those encoding oxidative stress defense enzymes, decreases, at least among certain cell types [52, 56]. Also, while *SOD1* is known to be essential for yeast longevity in liquid cultures [63, 64], it does not appear to be essential for colony survival and longevity [60, 61]. Cells in colonies are exposed to gradients of nutrients, waste products and gases whose complex spatial and temporal dynamics result in a mosaic of physiologically differentiated cell types that open up the possibility for myriad cell-cell interactions. Consequently, yeast growing as colonies on agar more closely resemble the tissues of multicellular organisms than do planktonic yeast in liquid culture [61]. Yeast growing as colonies might also be used to model mammalian cancer cells as both maintain high glycolytic flux; by contrast, starving planktonic cells may be a more a suitable model for tumor necrosis [56, 65].

U and L cells can be easily isolated [36] and their physiological differences exploited to model different types of metazoan cells. Whereas L cells could be used to model healthy mammalian tissue [56], U cells exhibit certain attributes of tumors, notably progressive changes in mitochondrial morphology such as swelling and loss of cristae [66], ammonia induced autophagy [67], lowered respiratory capacity [68], and the activation of amino acid biosynthesis and TOR [56, 69]. Further, nutrient and waste product flow between U and L cells are reminiscent of how the Cori and the glutamine-ammonium cycles interplay between healthy and tumor cells [51, 56]. Still, like starving planktonic yeast in liquid media, a yeast colony growing on agar is a closed system having limited material exchange with the external environment, save for gases or volatiles such as alcohols. In this respect, both techniques imperfectly model metazoan cells, which are open systems.

## CHRONOLOGICAL AGING IN CONTINUOUS CULTURE: THE RETENTOSTAT

### General considerations

In yeast, cell reproduction is usually coupled with metabolism [70]. Regardless of whether cultured as planktonic cells in liquid media, or as colonies on agar, yeast eventually ceases to divide because it lacks essential nutrients. By contrast, many animal cell types undergo G_0_ arrest in the presence of excess nutrients [71], and then begin to age chronologically. Another way to better model mammalian CLS with yeast is to culture it in a retentostat **(Figure 1C)**, a continuous-flow system whose operational principles were first described by Herbert [72]. This apparatus is a variant of the more familiar chemostat [73–77] where balanced growth of planktonic cells is achieved by continuous flow of a growth limiting-nutrient through a bioreactor. At steady state, microbial specific growth rate, *µ*, is equal to the dilution rate, D, defined as the outflow flow rate of spent medium (F_out_ in L/h) over the liquid volume of culture (VL) in the bioreactor. Depending on species, strain and type of limiting nutrient, chemostats are typically run at 0.4 h^-1^ > D > 0.03 h^-1^. Similar to chemostat, in a retentostat all nutrients save one, an energy source, are present in non-limiting concentrations. However, unlike a chemostat, cells in a retentostat are prevented from leaving the reactor in the spent medium stream. Thus, cells can be cultured to high cell densities (**Figure 1C**; ~15 g biomass per L) [78] at near-zero growth rates (D < 0.001 h^-1^). Continuous retentostat cultures maintain homeostasis between cells' rate of substrate consumption and their maintenance energy requirements. Like a chemostat, and indeed like a metazoan with a circulatory system, the retentostat is an open system, continuously exchanging nutrients and wastes with its external environment.

### Retentostat growth and viability

Boender *et al.* 2009 were among the first to study *S. cerevisiae* in retentostats. Under anaerobic conditions, in a chemostat running at D = 0.025 h^-1^, cells satisfy their maintenance energy requirements, estimated to be 0.50 mmol of glucose per gram of biomass per hour. Starting at D = 0.025 h^-1^, cell outflow can be blocked by filtration, transforming the chemostat into a retentostat. After 7 days, growth rate in the retentostat decreased to < 0.004 h^-1^, and after 22 days growth rate fell to < 0.001 h^-1^, corresponding to a doubling time of 27 days. Over 22 days of retentostat cultivation, cell viability fell from 91 ± 8% to 79 ± 6%. Glycogen content more than doubled over this interval, from 4.3 ± 0.8% in chemostat cultures at D = 0.025 h^-1^ to 9.1 ± 0.6% in retentostat cultures at 22 d (D < 0.001 h^-1^); trehalose content did not change (1.0 ± 0.4%). Retentostats therefore open up possibilities for studying cell physiology under conditions of severe CR and very low growth rate.

### Transcriptomics

Boender and colleagues have carried out genome-wide expression studies of retentostat yeast, comparing its profile to those of faster-growing chemostat yeast (D = 0.025 h^-1^) [79]. Beginning with a culture growth rate of 0.025 h^-1^ at day 0, growth rate in the retentostat decreased after 2 days to 0.0084 h^-1^, then leveled off at 0.00063 h^-1^ after 22 days [79]. At 22 days 15% of cells were budded, typical for non-growing *S. cerevisiae* [80], and viability was estimated to be ~80% [79]. Relative to chemostat-grown cells (D = 0.025 h^-1^), transcript levels in retentostat-grown cells (D = 0.00063 h^-1^) were increased for 615 genes, and decreased for 241 genes (*q*-value < 0.000188 by *K*-means clustering). Transcript levels of many house-keeping genes (e.g. *ACT1, PDA1, ALG9, TAF10, TFC1, UBC6)* did not differ between these two cultivation regimes [79], indicating sustained metabolic activity. Among genes whose transcript levels did increase relative to chemostat-grown cells, those related to mitochondrial function were strikingly over-represented, notably 32 of 76 genes encoding mitochondrial ribosomal proteins, as well as genes that encode respiratory chain sub-units (e.g. *ATP4, ATP7, ATP15, COX5B, COX8, COX9, COX11, COQ5, COQ9, SOC1, SCO2),* protein processing (*IMP1, IMP2, SOM1),* and mitochondrial membrane transport (*TIM17, TOM6*) [79]. Upregulation of mitochondrial functions may therefore represent specific cellular adaptations to near-zero growth unrelated to either oxygen tension or glucose concentration [79]. Of genes whose transcript levels decreased, those in lipid and sterol metabolism were over-represented, though this could be an artifact of amending anaerobic cultures with ergosterol and the oleate ester Tween-80.

Transcript levels were elevated for multiple genes involved in repairing damaged DNA or protein (*SIR2, RAD10, RAD24, RAD27*). Upregulation of *SIR2* and its homologs may enhance genome stability in retentostat-grown cells relative to cells in chemostats, as *SIR2* deletion is known to increase genome instability [81]. Diminished expression of *SIR2* homologs reduces longevity in planktonically-grown yeast [82, 83] and may impact human aging as well [84], making the behavior of these genes of particular interest.

### Proteomics

Calorically restricted retentostat yeast is metabolically active, and its gene expression program suggests it may be protected against DNA and/or protein damage, even after 22 days [85]. Given these observations, it is not surprising that the yeast proteome also changes upon induction of near-zero growth under CR [86]. Of 3,813 proteins detected in a study comparing retentostat cells to faster-growing cells (D = 0.025 h^-1^), the levels of 252 proteins significantly increased while an equal number decreased, altogether 13% of the detectable proteome. By comparison, 31% of the proteome is expressed differently between exponential and stationary phase batch cultures [87]. Among proteins whose expression increased relative to faster growing cells were those in the oxidative branch of the tricarboxylic acid (TCA) and glyoxylate cycles as well as 5 of 6 proteins in the succinate dehydrogenase complex (Sdh1p, Sdh2p, Sdh3p, Sdh1bp, and Shh3p) [88]; these proteins are typically not expressed under anaerobic conditions [89]. Among proteins whose expression decreased in retentostats were 4/17 proteins involved in ergosterol biosynthesis, and 8/14 proteins forming the Like-Sm ribonucleoprotein core [86], which is thought to activate mRNA decapping [90].

Interestingly, the transcriptome and proteome datasets correlated poorly, with only 146 of 504 proteins changing with the same sign and magnitude as their associated transcripts [86]. Of the 146 proteins whose changes were consistent with changes in their transcript levels, two-thirds were higher in retentostats, notably 28 of 110 of mitochondrial proteins and 11 of 53 proteins in oxidative phosphorylation. Poor correlation between transcript and protein levels has been previously observed in stationary phase cultures in *S. cerevisiae* and in *Schizosaccharomyces pombe* [91, 92]. This finding may stem from half-life differences between proteins (0.5 h < t_½_ < 20 h) [93] and mRNAs (3 min < t_½_ < 8 h) [94]. These differences may be exaggerated in cells under nutrient limitation as more energy is required to synthesize proteins (1957 mmol ATP/100 g formed biomass) than mRNAs (201 mmol ATP/100 g formed biomass) [95]. Transcriptomic and as well as proteomic datasets do indicate tight regulation over proteins and transcripts related to O_2_ consumption, suggesting that this is of special importance to calorically restricted cells.

### Starvation vs. caloric restriction

A key distinction between cells in retentostats and cells in nutrient-depleted planktonic cultures is that the former are not starving. Unrelieved, starvation inexorably leads to cellular deterioration [96–99]. By contrast, retentostat cells are calorically restricted, a condition operationally defined as cells being fed one third to one half that of cells fed *ad libitum*, without inducing malnutrition or starvation [13, 48, 86, 100]. In a retentostat cells are supplied with enough carbon and energy for cell maintenance and survival, but not enough for reproduction. But while they exhibit some features of starving cells, like increased heat shock resistance and expression of certain quiescence-related genes [79], calorically-restricted retentostat yeast are more metabolically active and have higher viability [14].

Starvation and CR are physiologically different states; this can be illustrated by inducing starvation in a retentostat via elimination of the sole carbon source. Calorically restricted retentostat yeast turn over ATP at a rate of 1 mmol per gram of biomass per hour [14]. By contrast, within 24 hours of starvation induction, ATP turnover rate falls to less than 2% of this value (0.013 mmol per gram of biomass per hour). Twenty-one days after starvation induction, ATP turnover rate drops even further, to 0.0002 mmol per gram of biomass per hour.

Compared to cells in chemostats (D ≅ 0.025 h^−1^), retentostat yeast shows decreased expression of genes required for protein synthesis [79, 86]. Still, there is evidence for protein synthesis [85] despite its energetic cost [101]. Active protein synthesis and recycling among such cells explains their high levels of metabolic activity (>70%) and viability (60%), even in 22-day old cultures [85, 86]. Although they are severely nutrient-limited, retentostat yeast is sensitive to further dietary restriction: starvation results in the immediate downregulation of a large fraction of the protein synthetic machinery (25%) [14, 85]. Twenty-six hours after starvation induction retentostat cells exhibit high levels of apoptosis (43%) and greatly diminished viability (15%).

To compare the transcriptome of starving cells to that of CR cells whose maintenance energy requirements are being met, Boender *et al.* cultured yeast in retentostats with and without glucose. 549 genes were differentially expressed [14]. Levels of 140 transcripts were upregulated in carbon starved cells, including genes encoding dehydrogenases (e.g. *MDH3, DLD1, BDH1, BDH2, SDH1*) involved in the glyoxylate, Cori, and TCA cycles, among others, as well as genes whose products are required for growth on non-fermentable carbon sources (e.g. *HBT1, FMP45, SPG4, SPG1*). Transcript levels for the remainder were downregulated in starved cells, including 109 genes in protein synthesis, as well as 30 genes involved in amino acid biosynthesis. Starvation and CR are thus distinct physiological states that can be expected to have very different impacts on cell lifespan.

### Yeast retentostats as a model for the study of chronological aging

Calorically restricted cells in retentostats can be used to gain fresh insight into factors governing lifespan in non-dividing metazoan cells. Unlike starving planktonic cells, retentostat cells do not differentiate into quiescent and non-quiescent cell types [102]; instead, cells remain in an extended G_1_ phase [103]. With its active metabolism and near-zero growth rate, retentostat yeast is well-suited to model non-proliferating metazoan cells that are poised in G_1_ and supplied with their maintenance energy requirements [104], including reserve (as opposed to active) epithelial stem cells [105] and cervical reserve cells [106]. Retentostat yeast could also be used to study disease, e.g., retentostat-cultured *rim15*Δ yeast exhibits phenotypes reminiscent of cancer cells, notably heat shock sensitivity [107] and unresponsiveness to anti-growth signals [108]. Still, retentostat cells are nutrient-limited, whereas many somatic metazoan cells are not. And even retentostat enthusiasts attest to the challenges of its set-up and operation [78, 109–111].

## CHRONOLOGICAL AGING IN CONTINUOUSLY-FED IMMOBILIZED CELL REACTORS

### General considerations

**Figure 3:**
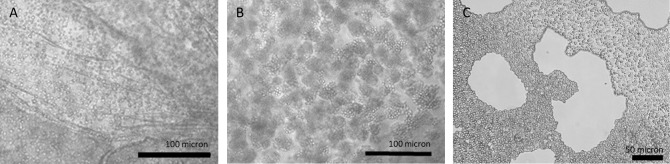
FIGURE 3: Yeast microcolonies form in alginate-encapsulated beads packed in immobilized cell reactors. Immediately after gelation (A), the interior of bead consists of primarily single, unbudded cells evenly dispersed throughout alginate matrix, but by 3 days in ICR (B), cells have evenly dispersed in clonal clusters of 4 or more cells. After 14 days in ICR (C), cells are tightly packed, occupying majority of matrix material. Hollow chambers are dispersed throughout beads, presumably formed by CO_2_ generation.

Microbial, plant and metazoan cells can be immobilized in a variety of matrices, ranging from naturally-derived hydrogels such as agarose, alginate, chitosan, collagen, fibrin, gelatin, and hyaluronic acid [112] to synthetic hydrated polymers and inorganic substrates, such as silica gels, sintered glass, and ceramic beads [113]. In naturally-derived hydrogels, microbial encapsulation (a type of immobilization) typically results in spherical beads whose diameter can be fixed in the range of micro- to millimeters. These beads, and the cells they contain **(Figure 3)**, can then be placed into immobilized cell reactors (ICRs; **Figure 1D)** and operated either as closed, batch-culture systems or as open, continuously-fed systems. The latter, which include stirred tank, packed and fixed-bed reactors [114], allows for continuous exchange of nutrients and waste between cells in their extracellular matrix and the liquid medium flowing through the reactor.

### Industrial applications

ICRs have chiefly been studied with an eye towards their use in biotechnology. There, they offer three advantages over conventional planktonic batch culture: (i) high productivity in terms of yield per cell, (ii) increased stability during the production cycle, and (iii) prolonged dry storage times. These advantages, reported by many [115–120], arise from the fact that gel-encapsulated cells reproduce slowly, if at all, and therefore divert little substrate to new biomass, which allows for more efficient substrate utilization [121]. Longer production cycles can be attributed to encapsulated cells' greater resistance to acids [122, 123], organic solvents [124, 125], ethanol [126], osmotic stress, and thermal stress [127, 128]. Greater stress resistance may also be associated with altered composition and organization of both the cell wall and plasma membrane [129], as well as with mechanical protection against shear provided by the encapsulating matrix [130]. In yeast, increased dry storage time without substantial loss in viability is consistent with the immobilized cells' higher content of storage and structural polysaccharides [116, 131, 132].

### Potential for cell-cell interactions among encapsulated yeast

Yeast cells immobilized in alginate ‘beads' cease to divide after reaching a certain density, which accounts for their high fermentative capacity, and likely also their exceptional longevity and resistance to stress. It is tempting to speculate that the physical proximity of such cells in a semi-solid matrix **(Figure 3)**, coupled with their entry into a G_0_-like state, confers upon them certain features of tissue level-organization, including social interactions. Strains isolated from the wild exhibit a variety of behaviors that have the potential to facilitate cell-cell interactions; these include floc [133] and flor formation [134], adhesion [135–137], as well as a kind of primitive multicellularity [138–141]. Yeast can also form biofilms [135], which in effect causes cells to become immobilized in a matrix of their own manufacture. Many of these behaviors are correlated with increased resistance to ethanol stress [142, 143], enhanced thermotolerance and osmotolerance [144], and improved survivorship in the presence of inhibitory compounds [145]. Similarly, encapsulated yeast become stress tolerant, though particular resistance profiles are specific to the type of encapsulating matrix [127, 146].

Social behavior in immobilized yeast appears to rely on intercellular signaling or quorum sensing (QS), which can be mediated by peptide pheromones [147] or by various organic compounds (alcohols, aldehydes and volatiles) [148]. An important criterion for a QS mediator is that it needs to reach a certain concentration before eliciting a concerted response which goes beyond that needed to metabolize or detoxify the compound [149]. One example of collective behavior in yeast is the onset of glycolytic oscillations relying on autocatalytic regulation of phosphofructokinase [150]. Glycolytic oscillations with a period of 25-32 s become synchronized in yeast cells depending on their concentration in alginate [151] and chitosan [152] beads, and are probably induced by locally high concentrations of acetaldehyde. We hypothesize that encapsulated yeast engage in social interactions and signal to each other in ways reminiscent of metazoan somatic cells [153]. Whether the G_0_-like state observed in such yeast is induced by QS, by mechanical forces like “self-jamming” [154], or is maintained by combination of chemical and mechanical factors remains to be explored.

### Physiology and transcriptomics

**Figure 4:**
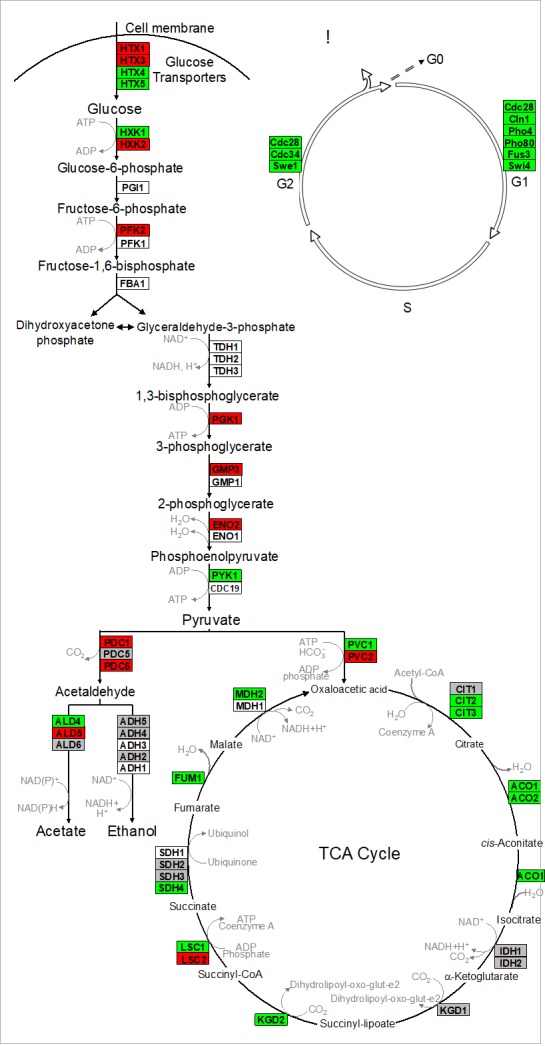
FIGURE 4: Alginate-encapsulated yeast cultured in continuously-fed bioreactors exhibit a stable pattern of gene expression in **(A)** intermediary metabolism, and **(B)** cell cycle where transcript abundance of glycolytic genes is increased, and that of TCA cycle and cell cycle genes is decreased. **Red** indicates genes where expression was at least two-fold greater in encapsulated cells than in planktonic cells over the course of 2 weeks culture. **Green** indicates instances where those values were at least two-fold less. **Gray** indicates no significant difference in sign or magnitude between planktonic and encapsulated cells (image adopted from Nagarajan *et al.* 2014 [132]).

Nagarajan *et al.* 2014 validated that reproduction is uncoupled from metabolism in continuously-fed, alginate encapsulated yeast, described one of its genetic determinants, and provided evidence that such cells are exceptionally long-lived [132]. The authors also showed that continuously-fed immobilized yeast exhibit a stable pattern of gene expression that is distinct from both growing and starving planktonic cells, consisting of an increased expression of genes in cell wall remodeling, glycolysis, and stress resistance, and an decreased expression of genes in the TCA cycle and cell cycle regulation (**Figure 4** and discussion below) [132]. These highly metabolically active cells achieved near-zero growth rates within 72 h post-encapsulation, and maintained this state for nearly 3 weeks, during which time upwards of 80% of cells remained virgin daughters [132]. Together, these attributes open up the possibility for developing a model to study cellular senescence and CLS in non-dividing eukaryotic cells, both in the absence and in the presence of CR.

Kruckeberg *et al.* developed methods to perform, for the first time, global analysis of gene expression in immobilized microbial cells [155]. Affymetrix GeneChips were used to profile the transcriptome of encapsulated yeast cells, compared to that of planktonic yeast grown either in glucose-limited chemostats or in glucose batch culture (mid-log and early stationary phase). Data from immobilized cultures revealed a pattern of gene expression that differed from planktonic cells, and remained stable over >2 weeks of continuous culture. Fewer than 100 genes changed by more than two-fold and none changed in sign. Transcript abundance conspicuously increased for multiple glycolytic genes (e.g., *HXK2, PFK2* and *PGK1*) and decreased for genes in the TCA cycle (e.g., *CIT2,3* and *ACO1,2*) and in the cell cycle (e.g., *CLN1* and *CDC28*) [156]. These data provide yet another line of evidence that ICR yeast is metabolically active, but growth-arrested.

Additional features of this dataset included increased transcription of genes that act in cell wall remodeling. *RPI1* up-regulation in encapsulated cells is especially noteworthy. Rpi1 acts as an antagonist to the RAS–cAMP pathway, and prepares yeast for entry into stationary phase by inducing transcription of genes whose products fortify the cell wall [157]. Like non-reproductive quiescent cells and spores, and like cells in the retentostat, immobilized yeast is highly heat-shock and zymolyase-resistant. However, unlike such cells, immobilized yeast fed *ad libitum* sustains a high rate of metabolism. In experiments described by Nagarajan *et al.* (2014), medium was continuously circulated within the bioreactor, exchanging the void volume once per minute. Every 48 h the feed reservoir was replaced with fresh SDC medium containing a five-fold excess of all micronutrients amended with 10% glucose as sole carbon source. Over 9 such cycles glucose was continuously fermented at near-theoretical yield with an undiminished rate of conversion.

Transcript levels of stress response regulators *MSN4* and *RIM15* were significantly increased in immobilized, but not in planktonic, cells. *RIM15* plays a role in cell cycle arrest, as multiple nutrient-sensing pathways converge on Rim15p [158]. *RIM15* has also been reported to promote chronological longevity in both starving planktonic yeast [100, 159] and yeast in retentostats [160]. Based on these converging lines of evidence, Nagarajan *et al.* hypothesized that this master regulator played a central role in uncoupling metabolism from reproduction in immobilized yeast. Flow cytometry of DNA content revealed that unlike wild type cells, *rim15*Δ cells exhibit a pronounced G_2_ peak at later time points in ICR culture, indicating that they continue to divide. After 5 days of continuous culture viability of immobilized *rim15*Δ yeast fell to 25%, compared to >90% in immobilized wild type cells. *RIM15* thus helps to mediate cell cycle arrest and stress resistance in the ICR model, and may contribute to ICR cells' extraordinary chronological longevity. The discovery that this remarkable physiological state is under genetic control opens the door to screening large numbers of barcoded knock-outs, a procedure likely to reveal new classes of longevity genes that alter lifespan in the absence of severe CR.

Of particular interest, but as yet not studied, is *XBP1,* which encodes a global regulator of entry into quiescent (Q) phase [161]. In this context, Xbp1 represses hundreds of genes following exhaustion of glucose, including G_1_ cyclins such as *CLN3* [162]. The Rad9/Rad53 checkpoint may act in the same pathway, protecting cells from replicative stress in cultures lacking glucose [163]. Similar to Q cells, recent data show that encapsulated cells are exceptionally resistant to starvation. Indeed, survivorship of starved encapsulated cells is several -fold higher than starved plank-tonic cells (Cook and Kroll, unpublished data). The XBP1/G_1_ cyclin pathway, in conjunction with starvation/stress signal transduction and replicative checkpoints, needs to be studied in encapsulated cells that cease to divide, similar to Q cells, but do so in the presence of nutrients, unlike Q cells. This may further connect well-fed encapsulated yeast cells to somatic cells in metazoans that also adjust their cell cycle machinery to uncouple reproduction from metabolism [164].

### Proteomics and metabolomics

Consistent with conclusions drawn from transcriptomics, a proteomic study of alginate-chitosan-encapsulated yeast in comparison with isogenic planktonic cells uncovered widespread changes in gene expression [146]. Encapsulated and planktonic cells were grown for 25 h in synthetic medium amended with 5% glucose, and the relative levels of 842 proteins were analyzed by mass spectrometry and 2D gel electrophoresis. A significant increase in central metabolic proteins was observed among immobilized cells, especially glutamine metabolism and fermentation, including proteins normally under glucose inhibition, such as high-affinity hexose transporters (Hxt6, Hxt7) and hexose kinases (Hxk, Glk1). This study also noted marked decreases in the level of proteins involved in protein synthesis and RNA transport. Interestingly, some stress response proteins were upregulated (Ssb2, Hsp12, Hsp26, Hsp78 and Hsp104), while others were downregulated (Ras2, Sod1, and many others) [146].

**Figure 5:**
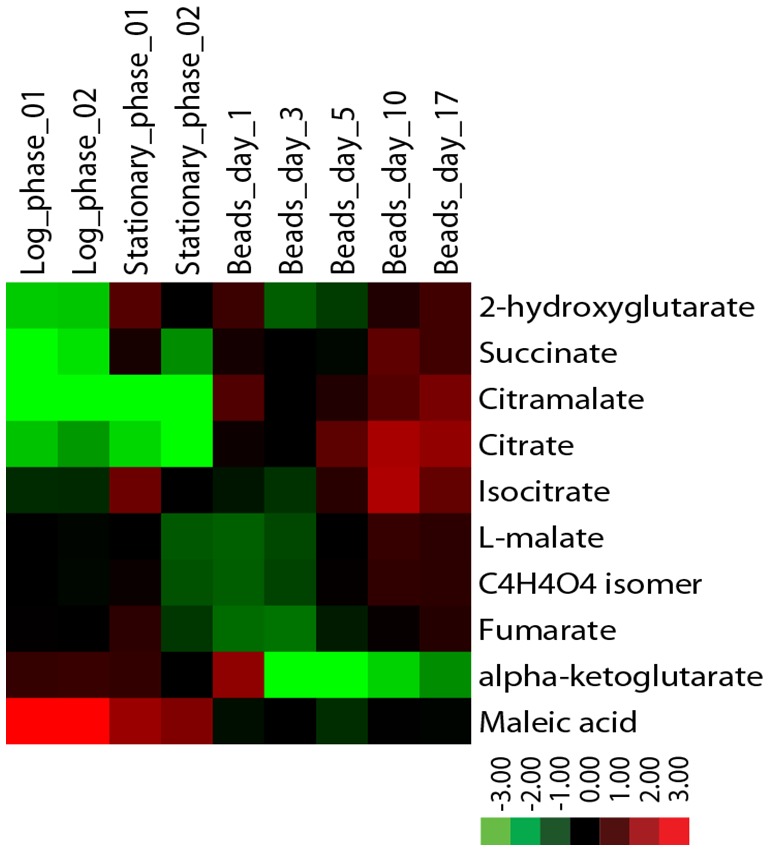
FIGURE 5: Levels of citric acid cycle metabolites in continuously-fed encapsulated yeast differ from planktonic yeast in exponential and stationary phases. Metabolites were extracted from flash-frozen cell pellets grown in batch or in continuous culture as indicated [172] and analyzed by LC-MS using a reversed phase method. Metabolite levels were normalized to the quantity of input cells, and displayed as a log2 fold change centered around the median value for each metabolite.

Metabolite levels also differ between encapsulated and planktonic culture conditions. When encapsulated cells are fed *ad libitum*, as in Nagarajan *et al.* [132], levels of succinate, citramalate, and citrate increase by several-fold relative to planktonic cells as determined by liquid chromatography mass-spectrometry **(Figure 5)**. By contrast, while levels of fumarate and malate are similar across all conditions, alpha-ketoglutarate levels decrease many-fold in encapsulated cells. Some of these metabolomic changes could not easily have been predicted from the transcriptomic data. For example, expression data reveal no significant differences between planktonic and encapsulated cells in the transcript levels of *IDH1/2* or *KGD1*, yet their respective substrate and product, alpha-ketoglutarate, is at very low levels in ICR. It is possible that such discrepancies can be explained, for example, by diminished expression of key enzymes upstream of a metabolite pool. In the case of alpha-ketoglutarate, expression of both citrate synthase (*CIT2/CIT3*) and aconitase (*ACO1/ACO2*) isoenzymes is significantly decreased in encapsulated cells relative to planktonic. Going forward, comparison of transcriptomic and metabolomic data obtained under identical conditions is sure to provide insight into biochemical changes that ensue following encapsulation, and further elucidate mechanism(s) by which encapsulated cells uncouple metabolism from cell division.

### ICRs for the identification of anti-aging compounds

Yeast microcolonies originating from a single cell are often used in high-throughput drug screens. As the measurement of colony size can be automated, this approach allows for large numbers of anti-aging compounds to be rapidly screened [165]. An obvious disadvantage of this approach is that it relies on cell division; hence, microcolony assays may fail to identify compounds that also extend CLS [104]. Because ICRs are populated with non-dividing, nutrient-replete cells, they offer the drug discovery process the advantage of directly assaying CLS. Though ICR-based assays are unlikely to be as easily multiplexed as those based on microcolony growth, or growth in liquid media, they could provide a valuable tool for testing whether anti-aging compounds uncovered by high-throughput assays also extend CLS.

### Encapsulated, continuously-fed yeast as a model for studying chronological lifespan

In metazoans, the great majority of cells exist in a non-dividing state, G_0_ [166–168]. While most are terminally-differentiated and will never divide, the lifespan of some G_0_ cells approximates the lifespan of the whole organism [169, 170], during which time they operate at full metabolic capacity [171]. Encapsulated yeast confined in ICR and fed *ad libitum* provides a reasonable approximation to post-mitotic metazoan cells like cardiac myocytes and neurons that are amply nourished but do not divide. Indeed, of all the zero-growth yeast models reviewed here, only the ICR model provides a way to study CLS in a simple eukaryote in the *absence* of CR, or starvation. This model could also be adapted for the study of CLS in the *presence* of CR simply by limiting carbon to levels that are one-third to one-half that of cells fed *ad libitum*, without inducing malnutrition or starvation [13, 48, 86, 100]. Systematic improvements that would enhance the utility of this model include: (1) multiplexing mini-ICRs so that individual columns could be sacrificially sampled over the course of weeks to months of continuous culture, (2) introducing mechanisms to sparge mini-ICRs so that CLS can be modeled under oxic and anoxic conditions, and (3) devising techniques to ensure even dispersal of diverse genotypes in a given bead, so that representatives of the barcoded yeast knock-out and overexpression collections could be studied under conditions of long-term growth arrest, either in the presence or absence of CR.

## CONCLUSIONS AND FUTURE DIRECTIONS

Genetic screens using the starving yeast model have provided insight into the behavior of eukaryotic cells under prolonged stress, showing how pathways such as TOR and genes like *SIR* are integrated and together contribute to cell survival. Because starving yeast are not dividing, this model has been used to measure CLS. As such cells are, by definition, nutrient-deprived, experimental results have been interpreted in the light of how CLS is affected by CR, a factor shown to extend longevity in virtually every species where it has been studied. But having few calories (CR) is not the same as having no calories (starvation). Thus, starving yeast does not mimic the physiology and lifespan of major classes of metazoan cells. Cardiac myocytes and neurons are terminally differentiated in a state of G_0_ arrest, but remain metabolically active and well-fed for the life of the organism. Muscle stem cells are also in G_0_-arrest and remain metabolically quiescent until recruited to enter G_1_ and produce cells that differentiate into, for example, myoblasts.

Alternative culture methods exist that enable yeast researchers to circumvent this fundamental problem. Yeast in giant colonies receive low levels of nutrients by diffusion, allowing them to divide over longer periods (~18 d vs. ~30 h) than starving planktonic cells. This longer period of slow growth contributes to the higher viability of aging cells in colonies relative to aging planktonic cells. Unlike starving planktonic cells, whose populations are not spatially structured, cells grown in colonies exhibit a primitive type of context-dependent cellular differentiation as they age, with some cell classes alive, but growing at near-zero rates. Ultimately though, yeast grown in giant colonies, like starving planktonic yeast, live in a closed, nutrient-depleted culture system that scarcely resembles a metazoan body.

Retentostats, an open system that exchanges nutrients and waste products with the environment, overcome several limitations associated with studying CLS in starving planktonic cells. Retentostat yeast is cultured at near-zero growth rates and provided with maintenance energy requirements. Retentostat yeast is under CR, a physiological state distinct from starvation. Cell viability remains high (80% after 22 days) with little evidence of protein and DNA damage. Aging retentostat yeast may therefore serve as a surrogate for classes of metazoan cells that have exited the replicative cycle such as pluripotent stem cells.

A third alternative to starving planktonic yeast is encapsulated yeast, which can be cultured in ICRs at near-zero growth rates. This matrix-associated, open culture system offers flexibility in nutrient supply. Yeast can be fed *ad libitum* so that non-dividing cells remain viable for weeks on end, conditions that mimic metabolism and longevity in terminally-differentiated metazoan cells. ICR yeast could also be cultured under nutrient-limiting conditions, making it possible to study cells in a tissue-like matrix under CR. Nutrient levels could even be reduced to where only maintenance energy requirements are met, creating an ICR model for the study of stem cells.

Caution should be exercised in the use of starving, planktonic microbial cells to evaluate determinants of CLS in metazoan stem cells and metazoans cells that are terminally differentiated. Since these cell types have their energy requirements met, alternative systems are needed that better mimic their physiologies. Yeast growing in colonies, in retentostats, and in immobilized cell reactors all provide a higher degree of biological realism.

## Abbreviations:

CFU: – colony forming unit,
CLS: – chronological lifespan,
CR: – caloric restriction,
ICR: – Immobilized cell reactor,
PCD: – programmed cell death,
Q: – quiescent,
QS: – quorum sensing,
RLS: – replicative lifespan,
TCA: – tricarboxylic acid,
TOR: – target of rapamycin.
